# Telemedicine Use among Adults with Kidney Disease in the United States

**DOI:** 10.34067/KID.0000000726

**Published:** 2025-02-12

**Authors:** Rishi M. Shah, Adhvaith Sridhar, Kavya M. Shah, Katie Stickels, Megan Crowther, Celine Kugler, Anthony Zhong, Li-Li Hsiao

**Affiliations:** 1 Department of Applied Mathematics, Yale College, New Haven, Connecticut; 2 Department of Biochemistry, Molecular Biology, and Biophysics, University of Minnesota Twin Cities, Minneapolis, Minnesota; 3 Department of Applied Mathematics and Theoretical Physics, University of Cambridge, Cambridge, United Kingdom; 4 Department of Biological, Physical, and Human Sciences, Freed-Hardeman University, Henderson, Tennessee; 5 Department of Nutrition, Dietetics, and Food Science, Brigham Young University, Provo, Utah; 6 Department of Biochemistry, Northeastern University, Boston, Massachusetts; 7 Harvard Medical School, Boston, Massachusetts; 8 Renal Division, Department of Medicine, Brigham and Women's Hospital, Boston, Massachusetts

**Keywords:** clinical epidemiology, kidney disease

## Introduction

The use of telemedicine has increased dramatically in recent years, with 37% of adults reporting having used telemedicine in 2021.^1^ Previous work has found that transportation barriers to health care disproportionately affect individuals with chronic conditions such as kidney disease.^2^ As such, individuals with kidney disease may particularly benefit from the use of telemedicine services as they can enable continuity of care while decreasing the burden of traveling to in-person appointments. However, little is known about telemedicine usage among those living with kidney disease. As telemedicine usage continues to rise, it is important to understand its effect on individuals living with kidney disease.

## Methods

We used data from the 2020 and 2021 editions of the National Health Interview Survey, a serial cross-sectional survey that produces a representative sample of the civilian, noninstitutionalized US population.^3^ Adult response rates in those years were 48.9% and 50.9%, respectively. Survey analysis was applied to obtain national estimates and correct for sampling and nonresponse bias. Adults aged 18 and older who self-reported kidney disease—defined as being diagnosed with weak or failing kidneys, excluding conditions such as kidney stones, bladder infections, or incontinence—were included in our analysis. Telemedicine use was defined as having had a medical appointment by video or phone in the past 12 months. A multivariable logistic regression model was constructed to investigate independent predictors of telemedicine usage. Statistical analyses were conducted in R version 4.3.1, with *P* < 0.05 being considered significant. We followed the Strengthening the Reporting of Observational Studies in Epidemiology guidelines. The Harvard University Institutional Review Board waived this study from review because National Health Interview Survey (NHIS) data are publicly available and deidentified. The NHIS is a serial cross-sectional survey with self-reporting by participants. Survey responses are not validated. The NHIS is administered by the Center for Disease Control and Prevention.

## Results

Our study sample consisted of 47,194 adult respondents, 1616 (3.42%) of whom reported having kidney disease. Among those self-reporting kidney disease, 954 (59.0%) reported having used telemedicine in the past 12 months, corresponding to a national estimate of 20.4 million (95% confidence interval [CI], 19.8 to 20.9 million) individuals with kidney disease who used telemedicine each year in 2020 and 2021. Of that group, 808 (84.7%), or an estimated 17.5 million (95% CI, 17.2 to 17.8 million), reported having used telemedicine because of reasons associated with the coronavirus disease 2019 (COVID-19) pandemic.

**Figure 1:**
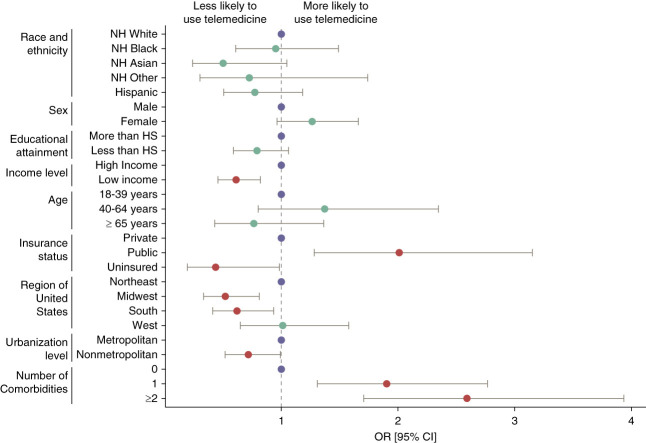
**Adjusted ORs of factors associated with telemedicine usage among US adults with self-reported kidney disease.** 24/47,194 (0.05%) and 30/47,194 (0.06%) of respondents refused to disclose or did not know their kidney disease status, respectively. An OR >1 indicates a higher likelihood of telemedicine use. Statistically significant ORs (Wald test *P* value < 0.05) are indicated in red, reference levels are indicated in purple, and all other ORs are indicated in green. CI, confidence interval; HS, high school; NH, non-Hispanic; OR, odds ratio.

From our multivariable regression model, we found that among those with kidney disease, those reporting public health insurance coverage (odds ratio [OR], 2.01; 95% CI, 1.1.28 to 3.15; *P* value [*P*] = 0.002) and those experiencing one (OR, 1.90; 95% CI, 1.31 to 2.77; *P* < 0.001) or two or more (OR, 2.59; 95% CI, 1.71 to 3.94; *P* < 0.001) comorbidities were more likely to use telemedicine. By contrast, individuals with kidney disease who were uninsured (OR, 0.44; 95% CI, 0.19 to 0.98; *P* = 0.046); lived in the Midwest (OR, 0.52; 95% CI, 0.33 to 0.81; *P* = 0.004) or Southern (OR, 0.62; 95% CI, 0.41 to 0.93; *P* = 0.023) regions of the United States; reported low income, defined as family income <200% of the federal poverty level (OR, 0.61; 95% CI, 0.46 to 0.82; *P* = 0.001); or lived in a nonmetropolitan area (OR, 0.72; 95% CI, 0.52 to 0.99; *P* = 0.045) were less likely to use telemedicine (Figure 1).

## Discussion

In this nationally representative study, over half of adults self-reporting kidney disease used telemedicine. We found that public insurance was a determinant of increased telemedicine use, likely because of expanded telemedicine coverage enacted by the US Centers for Medicare & Medicaid Services during the COVID-19 pandemic.^4^ Conversely, individuals with lower income, no insurance, and rural residence were less likely to use telemedicine, potentially because of financial barriers and limited access to technology and the Internet.^5^

These results suggest that bridging the digital divide is crucial to making telemedicine a more equitable form of care. Policymakers should consider initiatives such as subsidized Internet service, digital device distribution, technology-enabled community health centers, and rural broadband expansion, while health care providers should increase targeted education on telemedicine benefits because these initiatives may increase adoption.

We also found that age, sex, race, ethnicity, and educational attainment—factors typically linked to health care disparities in individuals with kidney disease—were not significant predictors of telemedicine usage.^6^ This could be caused by widespread reliance on telemedicine driven by COVID-19 restrictions, bolstered by broad cellular device ownership within the United States. While this result is encouraging, further observation is warranted to ensure these equitable patterns persist postpandemic.

This study has several limitations. First, the reliance on self-reported kidney disease diagnosis without clinical validation introduces potential misclassification bias; indeed, up to 90% of adults with mild CKD and more than 40% of those with moderate CKD are unaware of their condition, which may lead to underestimates in our analysis.^7^ Second, NHIS data on telemedicine use lacks granularity because it neither distinguishes between phone and video consultations, nor the purpose of a visit (*e.g*., medication management versus monitoring). This can make it difficult to assess how telemedicine use might vary with disease stage and patient needs. Finally, the rapidly evolving landscape of telemedicine expansion policies and reimbursement during the COVID-19 pandemic likely inflate estimates of telemedicine use and create complexities in interpreting trends. Nevertheless, by continually monitoring telemedicine use in the years following the pandemic, a clearer picture of its long-term effect on health care delivery for patients with kidney disease can be determined.

## Data Availability

All data are included in the manuscript and/or supporting information. Partial restrictions to the data and/or materials apply. Data are publicly available and can be accessed at https://www.cdc.gov/nchs/nhis/or through the Integrated Public Use Microdata Series Health Surveys website (https://nhis.ipums.org).
